# A commentary on: “Cortical and thalamic excitation mediate the multiphasic responses of striatal cholinergic interneurons to motivationally salient stimuli”

**DOI:** 10.3389/fncir.2015.00015

**Published:** 2015-04-14

**Authors:** Assunta Pelosi, Domenico Guarino

**Affiliations:** ^1^Neurotransmission and Signaling Lab, Institut du Fer à Moulin Institut National de la Santé Et de la Recherche Médicale UMR-S839, Université Pierre & Marie Curie, Sorbonne UniversitéParis, France; ^2^Neuroinformatics Group, Unit of Neuroscience Information and Complexity, Centre National de la Recherche Scientifique FRE 3293Gif-sur-Yvette, France

**Keywords:** basal ganglia, medium spiny neurons, striatum, salient stimuli, pause response, tonically active neurons, dopamine, acetylcholine

Each time there is an interaction with the environment, we select one among many, equally possible, actions. How is it done? In the classical view, to access motor resources, many cortical action plans compete in the basal ganglia: a set of subcortical neuron populations, whose output constantly inhibits specific thalamic targets. In the basal ganglia, one cortical plan is selected, the corresponding thalamic inhibition is disabled, finally producing the action (Albin et al., 1989). The classical view of action selection is now extended, including thalamic afferents. And, in the current view, a key role as modulators of action selection has been assigned to cholinergic interneurons of the striatum, the largest basal ganglia structure (Ding et al., 2010).

Striatum is divided in two major populations of GABAergic medium spiny neurons (MSNs) according to different expression of D1 and D2 dopamine receptors. These two populations and their downstream pathways are respectively addressed as direct and indirect, for their connections with output basal structures (Tepper and Bolam, 2004). More than 90% of striatal neurons are MSNs while the remaining 5–10% are two types of interneurons, cholinergic, and GABAergic. These are providing the lateral inhibition that can actually implement the competition among cortical plans and therefore are a key to understand action selection.

Cholinergic interneurons are known as tonically active neurons (TANs) for their spontaneous activity (>4 Hz). They receive glutamatergic afferents from cortex and thalamus, forming more synapses with thalamic than cortical terminals (Doig et al., 2014), and dopaminergic afferents from substantia nigra pars compacta. Locally, they branch dendritic arborization up to a millimeter in diameter, and they innervate GABAergic interneurons, other TANs and MSNs (Tepper and Bolam, 2004).

Under resting conditions, MSNs are hyperpolarized by TANs spontaneous tonic firing (Tepper and Bolam, 2004). Morris et al. (2004) showed that increased cortical activity makes TANs respond with a brief burst, an afterhyperpolarization pause in their firing, and a rebound burst. In addition, Ding et al. (2010) have shown, *in vitro*, that also thalamic stimuli elicit TAN burst-pause pattern response.

The work of Doig et al. (2014) aims at functionally distinguish thalamic and cortical contributions to action selection. Using different stimuli (single-, paired-pulse, and pulse trains) selectively delivered in the cortex and thalamus of rats, they were able to identify two temporal TAN response patterns. As in Morris et al. (2004), they used peri-stimulus time histogram (PSTH) analysis to identify the three main phases in TAN response: burst, pause and rebound. Applying pulse trains, which mimic the timing of motivationally salient stimuli (Ding et al., 2010), they were able to discriminate cortical and thalamic stimuli, predicting the whole TAN response based only on the initial phase. In fact, the number of spikes in the burst correlated positively with pause length and negatively with the magnitude of rebound: thalamic source was identified by high initial phase, long pause and low rebound, while cortical source was marked by lower initial phase, short pause, and higher rebound (Figure 1A).

**Figure 1:**
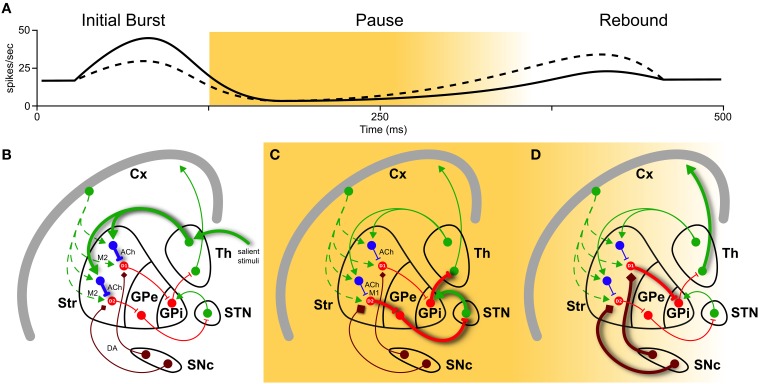
**Basal ganglia play a selecting role in motor activity**. To perform a certain movement, it is necessary to let motor thalamic neurons (Th) excite the cortex (Cx). But globus pallidus (pars interna, GPi) constantly inhibits them. The striatum (Str) is thought to encode action sequences whose occurrence could be facilitated or suppressed through direct and indirect pathways. Activation of the direct pathway, mediated by D1 receptors, as well as suppression of the indirect pathway, mediated by D2 receptors, (through globus pallidus pars externa, GPe, and sub-thalamic nucleus, STN) are therefore required to interrupt tonic pallidal inhibition and disinhibit the thalamic neurons, resulting in excitation of the cortex and the movement. Recently, an important role in modulation of basal ganglia responses has been attributed to striatal cholinergic TANs (in blue). They have different firing patterns depending on their inputs in **(A)**: a high initial burst, a long pause, and a low rebound burst when excited by thalamus (solid line), and a low initial burst, a shorter pause, and a higher rebound when excited by cortex (dashed line). These three phases have different impacts on action selection and reinforcement learning. **(B)** During burst induced by thalamic input (thick solid green), TANs reduce cortical input (dashed green) releasing ACh acting pre-synaptically on muscarinic receptors M2 (fast kinetic). **(C)** During the pause, muscarinic receptor M1 on D2 MSNs are slowly activated by ACh binding. This enhances responsiveness to corticostriatal input, actively braking any ongoing activity (thick solid red). **(D)** Still during the pause and the following rebound, a cortical plan is eventually selected and inhibits GPi through D1 pathway (thick solid red), unleashing an action. And if the action is rewarded, signaled by dopamine (DA) from substantia nigra pars compacta (SNc, thick brown), it creates ideal conditions for long-term modification of corticostriatal synapses, which is thought to underlie reinforcement motor learning.

To explore the contribution to action selection of these two different TANs responses, Doig et al. (2014) performed experiment in primates. The authors referred to the effect on striatum of salient stimuli, which are known to shift attention and suppress ongoing motor activity by engaging thalamostriatal-evoked pause in TANs (Desimone and Duncan, 1995). A reward-conditioning task was performed with macaque monkeys. Two conditions were used: reward-predicting visual stimulus, followed by juice delivery, and reward-only, in which juice was delivered randomly without any predictive stimulus. Although in these experiments the authors could not determine the (cortical or thalamic) origin of inputs, using their PSTH characterization, they found in monkeys a correlation similar to that seen in rats for thalamic-induced pattern and, more importantly, it was present only after reward-predicting stimuli and weak or absent after reward-only.

Taking into count these and other results in current literature, we might point a mechanism linking action selection (1) and salient stimuli (2) (see also Figure 1). The link is in the interplay of acetylcholine (ACh) and dopamine (DA) release, induced by thalamic-mediated salient stimuli.

1. TANs “thalamic” response pattern prepares the condition for action selection. In fact, after release, ACh binds to muscarinic receptors M2 (fast kinetic) and M1 (slow kinetic). M2 activation transiently reduces glutamate release from cortical terminals during the initial phase (Figure 1B). More effectively, M1 activation on D2 MSNs enhances their responsiveness to corticostriatal input throughout the pause (Ding et al., 2010, and Figure 1C). Hence MSNs located in proximity of pausing TANs will transiently become much more responsive to cortical input (Pakhotin and Bracci, 2007).
2. The same “thalamic” response pattern reinforces rewarded cortical actions. Its longer pause creates a window for a cascade of events: ACh, while suppressing the ongoing actions exciting D2 MSNs, induces local DA release from dopaminergic axons acting on ACh nicotinic receptors (Threlfell et al., 2012, Figure 1C). In addition, ascending dopamine neurons activity due to rewarded actions further increases DA levels (Figure 1D). Increase in DA will thus create ideal conditions for long-term modification of corticostriatal synapses, which is thought to underlie reinforcement motor learning (Pakhotin and Bracci, 2007).

In conclusion, Doig et al. (2014) contribute in clarifying the importance of cholinergic interneurons as modulators of action selection. With their work *in vivo*, they support the current view—hypothesized with *in vitro* data (Ding et al., 2010; Threlfell et al., 2012)—that ACh and DA work in synergy to select actions and reinforce learning.

## Conflict of interest statement

The authors declare that the research was conducted in the absence of any commercial or financial relationships that could be construed as a potential conflict of interest.
